# A Numerical Study of Geometry’s Impact on the Thermal and Mechanical Properties of Periodic Surface Structures

**DOI:** 10.3390/ma14020427

**Published:** 2021-01-16

**Authors:** Elzbieta Gawronska, Robert Dyja

**Affiliations:** Faculty of Mechanical Engineering and Computer Science, Czestochowa University of Technology, Dabrowskiego 69, 42-201 Czestochowa, Poland; robert.dyja@icis.pcz.pl

**Keywords:** mechanical properties of advanced materials, periodic surface structures, surface thickness, heat distribution, stress distribution, computer simulations, numerical modeling, modeling and simulation of material properties

## Abstract

The paper focuses on thermal and mechanical analysis of Periodic Surface Structure (PSS). PSS is a continuous surface with a specific topology that is mathematically formulated by geometric factors. Cubic P-surface (“primitive”), D-surface (“diamond”), and G-surface (“gyroid”) structures were simulated under load and heat transport using a numerical approach. We conducted our study by solving the stress and heat equations using the Finite Element Method (FEM). We achieved results using our software module, which generates PSS and simulates stress and temperature distribution. The stress model defined by dependence between stress and strain, gained from an experiment, and correlation of strain and displacement, gained from geometric conditions, was used in numerical experiments. The influence of geometric factors on the thermal and mechanical behavior of PSS was qualitatively determined. We showed decreasing effective stress values with an increased number of cells in the cubic domain for concerned PSS. It is important, because the increase in the number of cells does not increase the structure’s volume.

## 1. Introduction

Periodic Surface Structures (PSS), so-called Triply Periodic Minimal Surfaces (TPMS), have locally minimized surfaces. PSS and TPMS are the names used interchangeably in the paper. They entwine the 3D space and typically split the finite volume into tightly interwoven volume-filling domains with no curled closed voids (open structure). PSS show advantages in their structural and mechanical performance compared to conventional solid mass structures. Early medical applications used porous structures made of ceramic, salt-leached, and biomineralized. However, with the coming of additive manufacturing technology, the use of specially designed complex, minimal-surface-based porous microstructures for biomedical engineering has become a viable and profitable alternative. Attractive candidates with favorable properties are ensured by a large class of periodic minimal surface structures that define regular porous materials [1,2,3]. PSS is gaining more interest in the design community, including applications in high stiffness structures, impact energy dampers, chemical catalysts, and medical bone implants. More general thermal and mechanical applications utilize energy absorption, liquid permeability, heat transfer, stress distribution/deformation, or mechanical response in mining, aerospace, chemical industries, etc. [4,5]. Despite this interest, the characterization of the mechanical PSS response remains an open question.

Periodic surface structures are models with a minimum surface area that show periodicity in three independent directions in a three-dimensional space. The surface has a mean curvature of zero and can be periodically extended indefinitely in three directions. It can provide a concise description of many physical structures. The principle of minimizing the local area shows that symmetrical saddles are formed locally in TPMS, so each domain consists of one connected and infinite component [6]. As a matter of PSS’s high structural complexity, only additive manufacturing (AM) technologies can realize mathematically defined engineering scaffolding TPMS architectures using polymers or metal particles [7]. Structures derived from minimal surfaces and other related surfaces were conceived as lightweight construction materials as early as the 1970s [8]. Over time, these structures have been found to optimize competing properties such as stiffness and heat/energy transport. Such structures’ main advantage is the open-cell structure, which facilitates the complementary medium’s migration while maintaining a high degree of structural stiffness [9]. As a pore-forming unit, PSS can realize a digital representation of a porous structure. In [10], the authors modeled porous scaffolds for bionic bone using TPMS. Their research showed that the periodic surface structure gives an outstanding representation of bone density. By adjusting the TPMS function parameters, the model’s mechanical properties were adjusted and forced to close to the real bone model. The MIT (Massachusetts Institute of Technology) team designed one of the most durable light materials known and simulated three-dimensional PSS (G-surface) structures made of this material. Gyroid turned out to have remarkable geometrical and mechanical properties [11,12]. The tensile and compressive test results showed that the structure could be ten times stronger than steel but significantly lighter. Their findings showed that a crucial aspect of the tests performed has more to do with unusual geometric configurations than the material itself. Scientists proposed similar durable, lightweight structures made of different materials to create similar geometric features. PSS has no reflection symmetry or straight lines, which reduces the stress concentration effect in the structure and provides highly efficient mechanical properties compared to solid or lattice structures.

Emerging design opportunities are made possible by commercially reliable additive manufacturing technologies such as Selective Laser Melting (SLM) or Direct Metal Laser Sintering (DMLS). It allows the fabrication of high-resolution metal structures directly from computer-aided design (CAD) data converted to a stereolithography (STL) file. Such AM technologies in rapid prototyping radically increase the efficiency and profitability associated with the production of complex geometries [13].

Selective laser melting (SLM), one of the AM technologies, enables the fabrication of space-filling lattice structures with exceptional load-carrying capacity, customizable stiffness, controlled cell topology, cell size, and porosity. In work [14], the Schoen Gyroid (SG) unit cell, a periodic surface structure, was used to design the cell structures. The authors showed that the mechanical responses of cellular structures could be accurately described with FEM. The uniaxial strength modulus change with different printing directions, although the difference is relatively small. The selective laser melting technique produces more isotropic samples than those produced by other 3D printing techniques because SLM is a particle-based technique. It has been shown that the size of the TPMS sample affects its mechanical properties [15]. Heat-resistant load-bearing elements are common in airplanes and have high requirements for lightness and mechanical properties. In [16], the authors show that the lattice topology’s optimization allows for high mechanical properties and lightweight structures. The metal lattice structures showed excellent unidirectional load-carrying performance, and the porous structure of TPMS met the demands of multi-scale designs. All test specimens were produced in the technology of selective laser melting. The authors found that the sample with the periodic surface structure shows the best strength and stiffness properties of all the assessed structures, which is particularly useful for optimizing the lattice network’s topology in heat-resistant unidirectional supporting structures. Moreover, they also found that the primitive porous test specimens’ hardness showed a sudden increase in relative density (RD) due to their structural properties. In [17], the authors showed that TPMS structures have excellent mechanical properties due to isotropic, smooth surface transitions, and connectivity with open cells. Designing metal cellular structures with a triply periodic minimum surface area is a novel approach to lightweight and multi-functional structural applications. In [18], the authors investigated the mechanical properties and energy absorption capacity of three types of TPMS sheet structures (primitive, diamond, and gyroid) produced by selective laser melting (SLM) under compressive load. They classified the failure mechanisms of these structures and the printing accuracy using numerical analysis. Experimental results revealed the better stiffness and energy absorbing capacity of TPMS structures compared to body-centered cubic trusses. The finite element simulation results also showed that diamond and gyroid structures exhibit relatively uniform stress distributions in all grid cells subjected to compression, leading to stable collapse mechanisms and the desired energy absorption efficiency. In [19], the cubic periodic structure of various designs was simulated under quasi-static compression. For numerical tests and experimental verification, the authors used specimens fabricated by selective laser melting. The results identified that the surface thickness and number of cells strongly influence compressive strength. These observations affected the theoretical development of a Gyroid structure that imitates both elastic and compressive strength modulus human cortical bone. In [20], the authors investigated the effect of cellular structure on stiffness and strength by comparing the behavior of np-Au structures with spinodal and gyroid structures. The results showed that the macroscopic stiffness and strength were highly sensitive to the topology genus. The topology effects were captured into modified scaling laws where the geometric pre-factors for the stiffness and strength are linearly dependent on the scaled genus density.

In this article, numerical simulations are made with properties for a sample material Al2% Cu alloy and verified for the full-size structure’s mechanical and thermal response. Based on this numerical model, the influence of the identified geometrical factors: surface thickness, sample size, number of surface periods, or unit cells was investigated using the authors’ software. In the paper, we used periodic minimal surface structures, maintaining the same, regardless of the direction in which they are measured, mechanical properties in all directions. Lack of differences is beneficial due to physical properties such as thermal expansion, thermal or electrical conductivity, refractive index, etc. We simulated the cubic P-surface, the D-surface, and the G-surface structures with various geometry factors under load and heat transport using a numerical approach. It is critical to know how new materials, structures, or models behave under load. Therefore, many researchers, including us, study the obtained results of the finite element methods (FEM) analysis of stress and strain distributions in order to approximate the mechanical parameters of devices [21,22,23]. In this way, the models become useful for the comparative analysis of different geometries, which—together with appropriate empirical validation studies—allow functional structure development. The influence of geometric factors on thermal and mechanical behavior was qualitatively determined.

The article deals with the thermal and mechanical analysis of periodic surface structures. The results showed the effect of cells’ number and surface thickness on both moduli. The simulations were carried out by solving the stress and heat equations using the finite element method (FEM) using our own code (implemented in C++). Our software module generated periodic surface structures and simulated the stress and temperature distribution in them. The forces acting on the element increased the stresses in it. The accumulation of stresses may cause local exceeding of the yield point and uncontrolled deformation of the element, and in extreme cases, breaking the continuity of the material. We consider the case of a static load. According to the referencing literature’s tests and experiments, our simulations showed the close relationship of stress distribution and temperature on geometrical factors.

## 2. Methods

The literature proved that the mean curvature of a minimal surface is zero at every point. Any infinitesimal region of such a surface has the least area of any region with the same boundary conditions [24]. Furthermore, the divergence of the unit normal vector n is zero throughout the minimal surface [25]. Periodic surface structures satisfy minimal surfaces’ requirements while self-tessellating infinitely in three mutually perpendicular coordinate directions [26]. In local Cartesian coordinates (X, Y, Z), we consider three mathematically defined periodic surface structures, the primitive (P), diamond (D), and gyroid (G) structure:

(1) (P) cosX+cosY+cosZ=δ

(2) (D) cosZsin(X+Y)+sinZcos(X−Y)=δ

(3) (G) sinXcosY+sinYcosZ+cosXsinZ=δ

where relative thickness *δ* is normalized, takes values from 0 to 1, and plays a significant role in the PSS topology and associated structural response. These implicit surfaces are defined as an isosurface of some function *f*:

(4) f(X, Y, Z)=0

PSS structural behavior’s computer simulation is based on efficiently generated, implicitly defined, and rapidly constructed finite element meshes. Gmsh software answered for generating finite element mesh for the bulk. Unfortunately, it did not have a design NURBS (non-uniform rational basis spline) surface, so mesh for PSS was created with our software using the CGAL (computational geometry algorithms library) library, which contains methods for describing the division of an area into finite elements. CGAL is a project that supplies easy access to efficient and reliable geometric algorithms in a C++ library. CGAL is used in various areas that need geometric computation, such as geographic information systems, computer-aided design, medical imaging, computer graphics, and robotics [27,28,29,30].

Heat transfer simulation was based on the heat transfer equation:

(5) $$\rho c\dot{T}-k\nabla^{2}T=0$$

where *ρ* is density, *c* is specific heat capacity, *T* is temperature, and *k* is thermal conductivity. The Neumann boundary condition is used for the description of heat flow into a calculation domain.

Stress analysis is a general term used to describe the quantities of stress and strains. It is also known as structural analysis. The most crucial parameter concerning many structures is the elastic modulus and yield strength [31]. Increasing Young’s modulus ensures improved mechanical properties necessary for counteracting high loading conditions [32]. The dependency of stress and strain characterizes the stress model obtained from an experiment and the association of strain and displacement obtained from geometric considerations. Stress is related to yield through the physical connection:

(6) σ=Cϵ

where *σ* is a stress tensor, *C* is a stiffness tensor, and *ϵ* is a tensor of elastic deformation. Physical properties that appear in the elasticity tensor can depend on temperature. The relationship from Equation (6) is called a generalized Hook’s law. In turn, the strain is related to displacement through the Cauchy relations [33]:

(7) $$\epsilon =\frac{1}{2}\left[\left(\nabla q\right)+{\left(\nabla q\right)}^{T}\right]$$

where *q* is the displacement vector. This work focuses on stress distribution in the three-dimensional case, so the stiffness tensor becomes:

(8) $$\left[\begin{array}{cccccc} \lambda +2\mu & \lambda & \lambda & 0 & 0 & 0\\ \lambda & \lambda +2\mu & \lambda & 0 & 0 & 0\\ \lambda & \lambda & \lambda +2\mu & 0 & 0 & 0\\ 0 & 0 & 0 & \mu & 0 & 0\\ 0 & 0 & 0 & 0 & \mu & 0\\ 0 & 0 & 0 & 0 & 0 & \mu \end{array}\right]$$

where

(9) $$\lambda =\frac{E\upsilon }{\left(1+\upsilon \right)\left(1-2\upsilon \right)}$$

(10) $$\mu =\frac{E}{2\left(1+\upsilon \right)}$$

and *E* is Young’s modulus, *υ* is Poisson’s ratio. In this case, the stress tensor has the following structure:

(11) $$\sigma^{T}=\left\{\sigma_{x}\sigma_{y}\sigma_{z}\tau_{xy}\tau_{xz}\tau_{yz}\right\}.$$

Meanwhile, the tensor of elastic deformation is given by:

(12) $$\epsilon^{T}=\left\{\epsilon_{x}\epsilon_{y}\epsilon_{z}\gamma_{xy}\gamma_{xz}\gamma_{yz}\right\}$$

where $\gamma_{xy}=2\;\epsilon_{xy}$. Furthermore, the displacement vector is equal:

(13) $$q^{T}=\left\{u\;\upsilon \;w\right\}$$

where x, υ, w are displacements in the direction of X, Y, Z axes, respectively.

## 3. Numerical Simulations Results and Discussion 

The article presents the heat conduction and stress distribution simulations for a cube that has a 0.04 m edge length and the periodic minimal surface structures inserted into it. Changing the number of unit cells does not change the size of the cube. Properties used in simulations are as follows: *k*—heat transfer coefficient 260 W/(m·K), *ρ*—density 2800 kg/m^3^, *c*—specific heat capacity 1000 J/(kg·K). The material properties are similar to those of aluminum–copper alloys—materials that are significant and often used in the industry. Particularly, aluminum alloys (AlSi, AlCu, AlSiMg, and others) are widely used in additive manufacturing. Authors considered the properties of sample materials Al2%Cu alloy because of their previous research of thermo-mechanics response during solidifying. On one of the boundaries (on the left side), the Neumann boundary condition with a fixed value of 10 kW/m^2^ was prescribed. All other five sides were implicitly set to a no-flux boundary condition (perfect insulation). It was assumed that there is no heat exchange with the environment, and the initial temperature was equal to 300 K. Mechanical properties are as follows: Young’s modulus *E* = 6.9 × 10^10^ MPa, Poisson’s ratio *υ* = 0.33, density *ρ* = 2700 kg/m^3^. All cubes had fixed bottom faces (all degrees of freedom were removed), and all cubes were subject to a load of value 10 MPa on the top face. With this value of the load, all investigated domains remained in the elastic region.

Table 1 presents different parameters used for generating the PSS, together with their volume and side area fractions calculated as a ratio of value for a given structure to volume or side area of the full cube. It can be seen that the number of cells for a given structure does not impact volume ratio; only thickness can change volume fraction. However, in the case of side area fraction, both the number of cells and relative thickness affect this indicator, but thickness still has a much more visible impact.

All periodic surfaces and the cube were discretized with linear tetrahedral elements. Details about meshes for presented results are gathered in Table 2.

**Figure 1:**
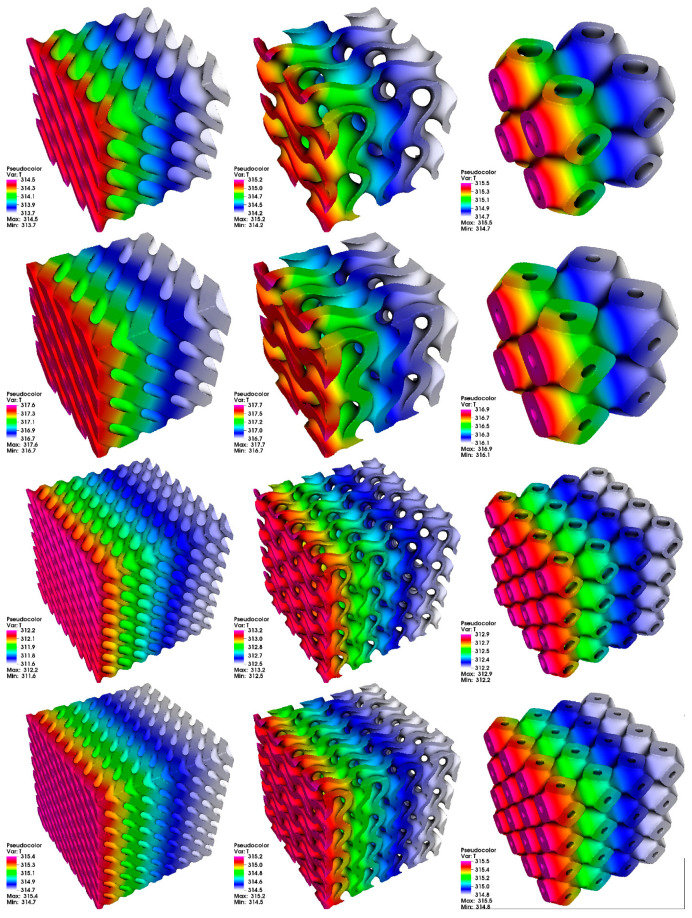
The results of heat simulation after 250 s. Columns from left to right: diamond (D) surface, gyroid (G) surface, primitive (P) surface. Rows from top to bottom: 2 cells, thickness δ = 0.125; 2 cells, thickness δ = 0.2; 4 cells, thickness δ = 0.125; 4 cells, thickness δ = 0.2

The heat simulation results are presented in Figure 1, where temperature distribution is shown after 250 s of simulation time. Heat simulation with the imposed boundary conditions described earlier will not achieve a steady state, so a point in time was chosen far away from initial rapid changes of temperature under prescribed heat flux. It can be seen that the PSS in all configurations characterizes by regular temperature distribution, and there are no overheated regions. Maximum achieved temperature is varied for different TPMS and different configurations, and this is because of the different volume that each PSS has (it is also affected by the relative thickness δ). Since heat flux on the boundary had constant density, the amount of energy income into material was greatly affected by the boundary’s surface area. 

The results from heat transfer simulation are summarized in Table 3.

**Figure 2:**
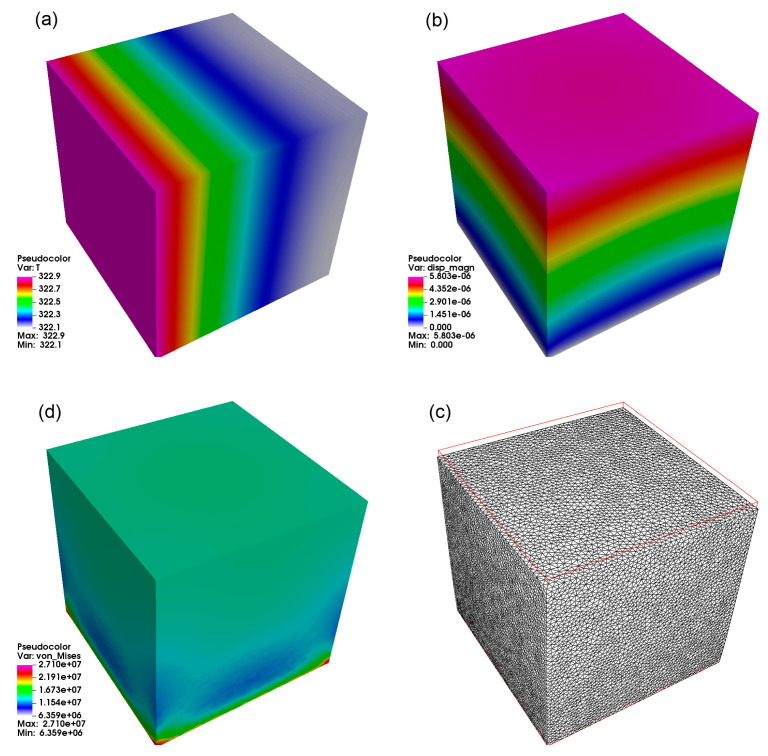
Results for a reference cube. The following result from the upper-left clockwise results: (**a**) heat distribution after 250 s, (**b**) total displacement from stress analysis, (**c**) mesh deformation with scale ×250, and (**d**) von Mises stress distribution in MPa.

The most remarkable difference can be seen if we compare the reference cube results (Figure 2) with the PSS. While the cube had the greatest volume (resulting in greatest heat capacity—all simulations were performed with the same material), it also had the greatest surface area, which resulted in the highest influx of heat to the domain. This is why the cube has the highest maximum temperature (in the range 322 K), comparing to the PSS (which had the maximum temperature in the range 312−317 K).

**Figure 3:**
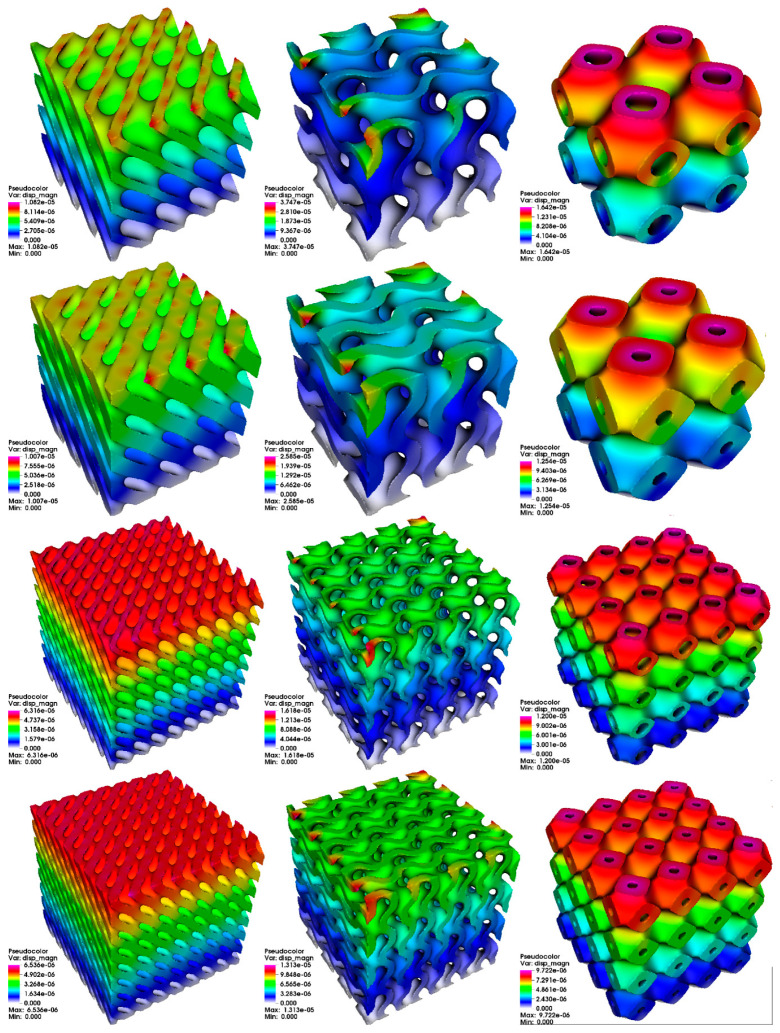
The results of total displacement from stress analysis. Columns from left to right: diamond (D) surface, gyroid (G) surface, primitive (P) surface. Rows from top to bottom: 2 cells, relative thickness δ = 0.125; 2 cells, relative thickness δ = 0.2; 4 cells, relative thickness δ = 0.125; 4 cells, relative thickness δ = 0.2.

**Figure 4:**
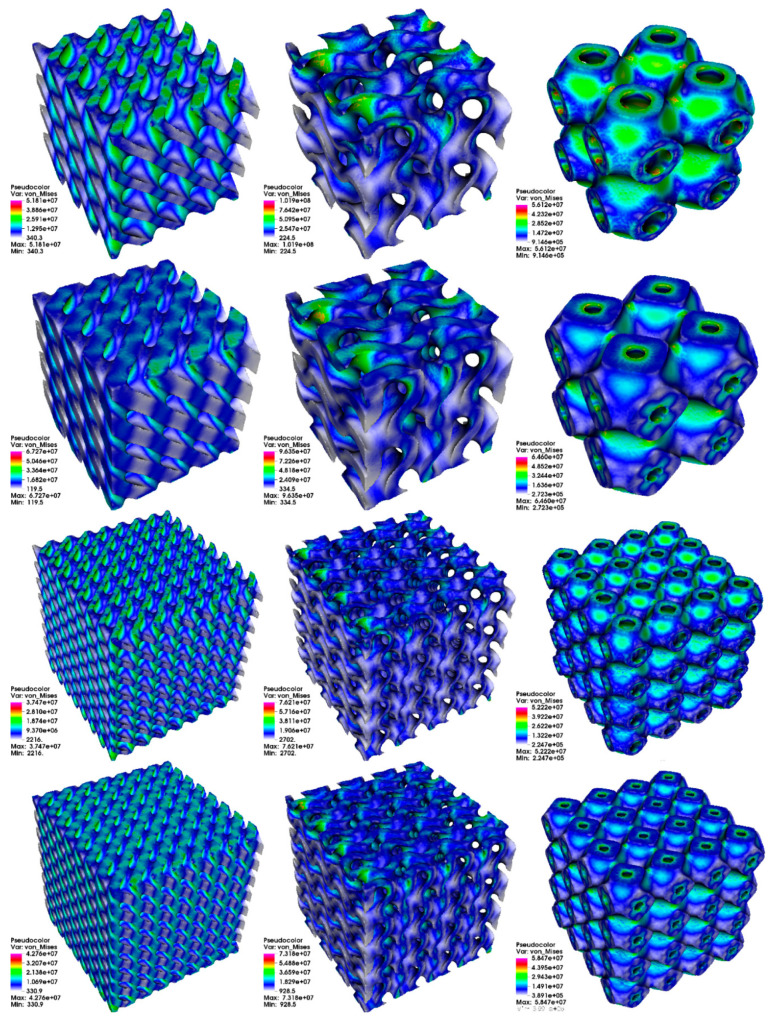
Von Mises stress distribution in MPa. Columns from left to right: diamond (D) surface, gyroid (G) surface, primitive (P) surface. Rows from top to bottom: 2 cells, relative thickness δ = 0.125; 2 cells, relative thickness δ = 0.2; 4 cells, relative thickness δ = 0.125; 4 cells, relative thickness δ = 0.2.

**Figure 5:**
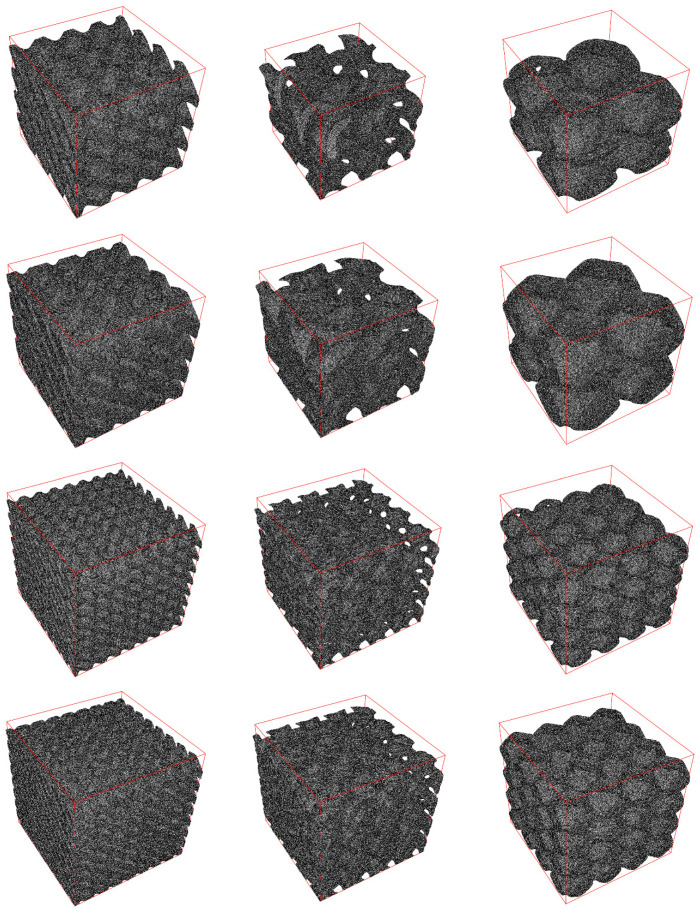
Mesh deformation. Columns from left to right: diamond (D) surface, gyroid (G) surface, primitive (P) surface. Rows from top to bottom: 2 cells, relative thickness δ = 0.125; 2 cells, relative thickness δ = 0.2; 4 cells, relative thickness δ = 0.125; 4 cells, relative thickness δ = 0.2.

The results for stress analysis are presented in Figure 3 (total displacement), Figure 4 (von Mises stress distribution), and Figure 5 (deformation of mesh). It can be seen here that the type of PSS has a visible impact on total displacement. Moreover, for types D and G, an increase in the number of cells in cube lowers the maximum total displacement. For type P, the number of cells has less visible impact. We can also see that increasing thickness can affect total displacement. Interestingly, we can observe that type D and G have some fragments in the structure that do not participate in stress propagation for the stress distribution. In contrast, for type P, all fragments participate. It results in a much more uniform stress distribution for the type P structure. 

The results from stress analysis are summarized in Table 4.

## 4. Conclusions

The paper presented different periodic surface structures and their behavior in engineering calculations. The authors also explored the potential influence of such factors as relative thickness or number of cells per unit cube on the results of simulations. Based on the paper results, it can be observed that all structures presented in the paper can be interesting for engineers planning to use them. However, eventual use should be preceded by careful analysis, because they have different properties.

The authors especially want to point out that there are differences in investigated structures in terms of stress distribution in the case of stress analysis. While for the primitive (P) surface, all parts of the domain participate in stress distribution, diamond (D) and gyroid (G) surfaces have some volumes with very low effective stress values. It suggests that they do not contribute to the overall strength of a structure. An additional conclusion is that we observe the effect of decreasing effective stress values with the increased number of cells in the domain for all surfaces. However, the exact values depend on a specific PSS. It is important because an increase in the number of cells does not increase the structure’s volume. We can observe reduced maximum displacement for periodic surfaces with increased relative thickness based on results obtained from stress analysis. It is in general conformity with scale law that governs such structures’ mechanical properties, including the Young modulus. Unfortunately, with only two relative thicknesses available for each structure, it is difficult to identify the exact character of this relation. In [34], Downing et al. present stress analysis for gyroid structures made from Ti6Al4V alloy. Downing et al. conducted a study of the influence of thickness and material distribution on TPMS stress level under compression. As mentioned in the paper, the von Mises stress magnitude plot showed a very similar pattern to the authors’ results. Additionally, [35] by E. H. Khogalia et al. shows stress analysis results for different TPMS variants under compressive loading. That study aimed to assess the performance of TPMS as a substitute of tissue material that can be used in prosthetic devices, and it also presents a trend of visibly better stiffness with an increased relative thickness that can be visible in Table 4.

Based on the results from heat simulation, it can be observed that all structures give a very uniform temperature gradient, which is very similar to the temperature gradient observed in the full cube. In fact, all PSS have a temperature difference between a coldest and hottest point in range ΔT = 0.6 K up to ΔT = 1.0 K, while a full cube has ΔT = 0.8 K. It shows that all PSS do not have any local hot points and can be useful in scenarios where PSS is subject to one-side heating.

## Figures and Tables

**Table 1:** Parameters used for generating different periodic surface structures, together with their impact on volume and the side area of a specific structure.

Name	Relative Thickness δ [.]	Thickness [m]	Volume [m^3^]	Volume Fraction [.]	Side Area [m^2^]	Side Area Fraction [.]
cube	-	-	6.400 × 10^−5^	1.0000	16.000 × 10^−4^	1.0000
diamond (D) 2 cells	0.125	0.0023	2.633 × 10^−5^	0.4114	4.385 × 10^−4^	0.2741
diamond (D) 2 cells	0.2	0.0041	4.254 × 10^−5^	0.6647	8.389 × 10^-4^	0.5243
diamond (D) 4 cells	0.125	0.0011	2.638 × 10^−5^	0.4122	3.555 × 10^−4^	0.2222
diamond (D) 4 cells	0.2	0.0020	4.266 × 10^−5^	0.6666	7.536 × 10^−4^	0.4710
gyroid (G) 2 cells	0.125	0.0023	2.069 × 10^−5^	0.3233	3.603 × 10^−4^	0.2252
gyroid (G) 2 cells	0.2	0.0040	3.343 × 10^−5^	0.5223	6.603 × 10^−4^	0.4127
gyroid (G) 4 cells	0.125	0.0012	2.071 × 10^−5^	0.3235	2.892 × 10^−4^	0.1808
gyroid (G) 4 cells	0.2	0.0020	3.347 × 10^−5^	0.5230	5.876 × 10^−4^	0.3673
primitive (P) 2 cells	0.125	0.0028	1.828 × 10^−5^	0.2856	3.138 × 10^−4^	0.1961
Primitive (P) 2 cells	0.2	0.0045	2.930 × 10^−5^	0.4579	5.545 × 10^−4^	0.3466
primitive (P) 4 cells	0.125	0.0012	1.829 × 10^−5^	0.2857	2.733 × 10^−4^	0.1708
primitive (P) 4 cells	0.2	0.0024	2.933 × 10^−5^	0.4584	5.096 × 10^−4^	0.3185

**Table 2:** Details of finite element meshes used for obtaining presented results.

Name	Relative Thickness [.]	Number of Nodes	Number of Elements
cube	-	45,142	245,979
diamond (D) 2 cells	0.125	325,910	1,087,687
diamond (D) 2 cells	0.2	322,198	1,122,255
diamond (D) 4 cells	0.125	551,898	1,731,035
diamond (D) 4 cells	0.2	544,391	1,911,853
gyroid (G) 2 cells	0.125	269,511	890,282
gyroid (G) 2 cells	0.2	283,175	967,104
gyroid (G) 4 cells	0.125	457,834	1,410,877
gyroid (G) 4 cells	0.2	479,653	1,610,759
primitive (P) 2 cells	0.125	211,320	706,578
Primitive (P) 2 cells	0.2	224,711	775,721
primitive (P) 4 cells	0.125	355,299	1,096,937
primitive (P) 4 cells	0.2	378,416	1,274,439

**Table 3:** Maximum, minimum, and temperature difference from heat transfer simulation.

Name	Relative Thickness [.]	Minimum Temperature [K]	Maximum Temperature [K]	Temperature Difference ΔT [K]
cube	-	322.1	322.9	0.8
diamond (D) 2 cells	0.125	313.7	314.5	0.8
diamond (D) 2 cells	0.2	316.7	317.6	0.9
diamond (D) 4 cells	0.125	311.6	312.2	0.6
diamond (D) 4 cells	0.2	314.7	315.4	0.7
gyroid (G) 2 cells	0.125	314.2	315.2	1.0
gyroid (G) 2 cells	0.2	316.7	317.7	1.0
gyroid (G) 4 cells	0.125	312.5	313.2	0.7
gyroid (G) 4 cells	0.2	314.5	315.2	0.7
primitive (P) 2 cells	0.125	314.7	315.5	0.8
Primitive (P) 2 cells	0.2	316.1	316.9	0.8
primitive (P) 4 cells	0.125	312.2	312.9	0.7
primitive (P) 4 cells	0.2	314.8	315.5	0.7

**Table 4:** Maximum total displacement, maximum and minimum von Mises stress from elastic stress analysis.

Name	Relative Thickness [.]	Maximum Total Displacement [m]	Minimum von Mises Stress [MPa]	Maximum von Mises Stress [MPa]
cube	-	5.803 × 10^6^	6.359 × 10^6^	2.710 × 10^7^
diamond (D) 2 cells	0.125	1.062 × 10^5^	340.3	5.161 × 10^7^
diamond (D) 2 cells	0.2	1.007 × 10^5^	119.5	6.727 × 10^7^
diamond (D) 4 cells	0.125	6.316 × 10^6^	2216	3.747 × 10^7^
diamond (D) 4 cells	0.2	6.536 × 10^6^	330.9	4.276 × 10^7^
gyroid (G) 2 cells	0.125	3.747 × 10^5^	224.5	1.019 × 10^8^
gyroid (G) 2 cells	0.2	2.585 × 10^5^	334.5	9.635 × 10^7^
gyroid (G) 4 cells	0.125	1.618 × 10^5^	2702	7.621 × 10^7^
gyroid (G) 4 cells	0.2	1.313 × 10^5^	928.5	7.318 × 10^7^
primitive (P) 2 cells	0.125	1.642 × 10^5^	9.146 × 10^5^	5.612 × 10^7^
Primitive (P) 2 cells	0.2	1.254 × 10^5^	2.723 × 10^5^	6.460 × 10^7^
primitive (P) 4 cells	0.125	1.200 × 10^5^	2.247 × 10^5^	5.222 × 10^7^
primitive (P) 4 cells	0.2	9.722 × 10^6^	3.891 × 10^5^	5.847 × 10^7^

## Data Availability

Data is contained within the article.
